# Supplementary figures and images for: Sleep and cognition in South African patients with non-functioning pituitary adenomas
Source: PLoS One. 2024 Jan 18;19(1):e0296387. doi: 10.1371/journal.pone.0296387 (PMC10796019; doi:10.1371/journal.pone.0296387)

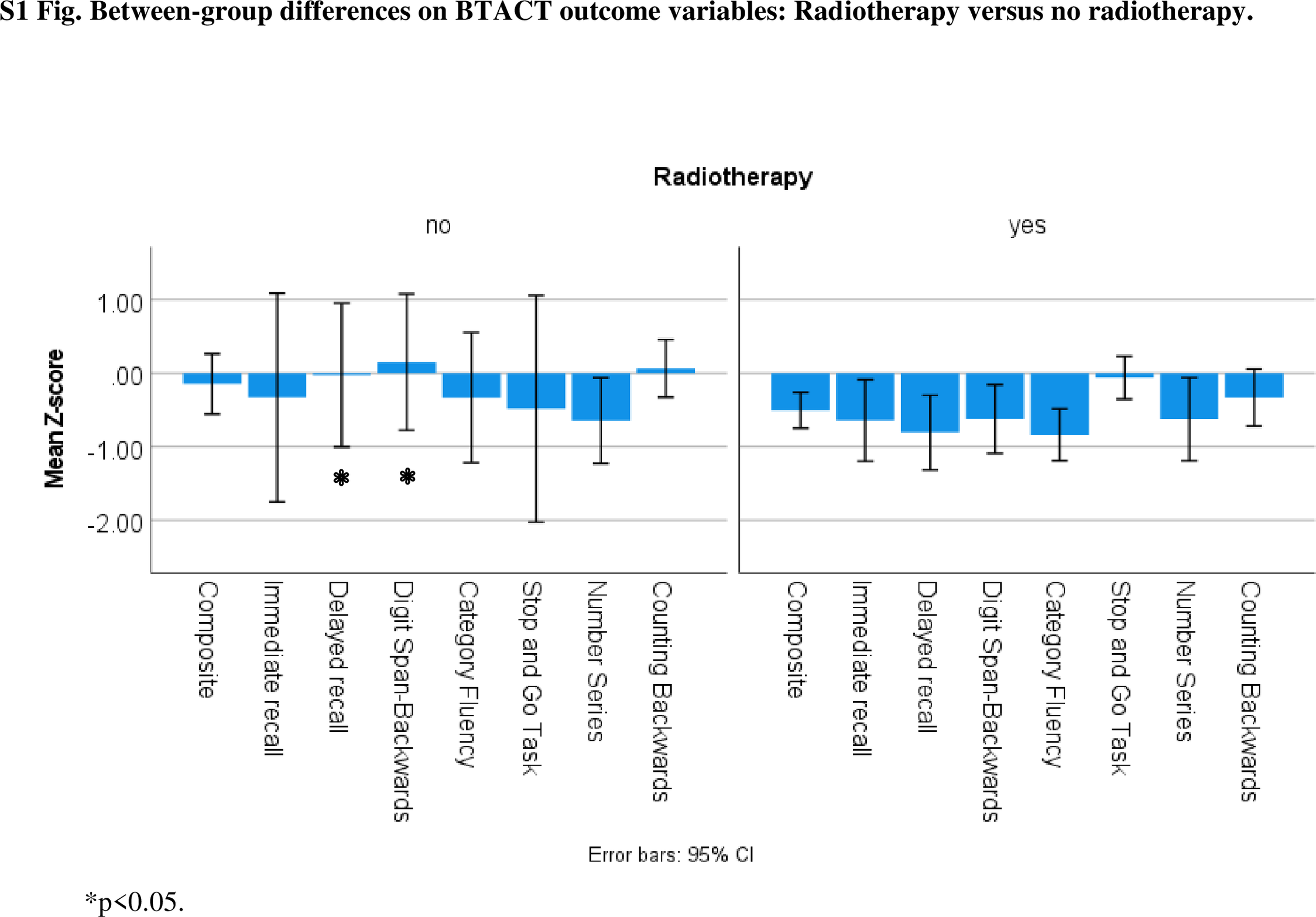

Supplement: S1 Fig — (TIF) [file pone.0296387.s001.tif]

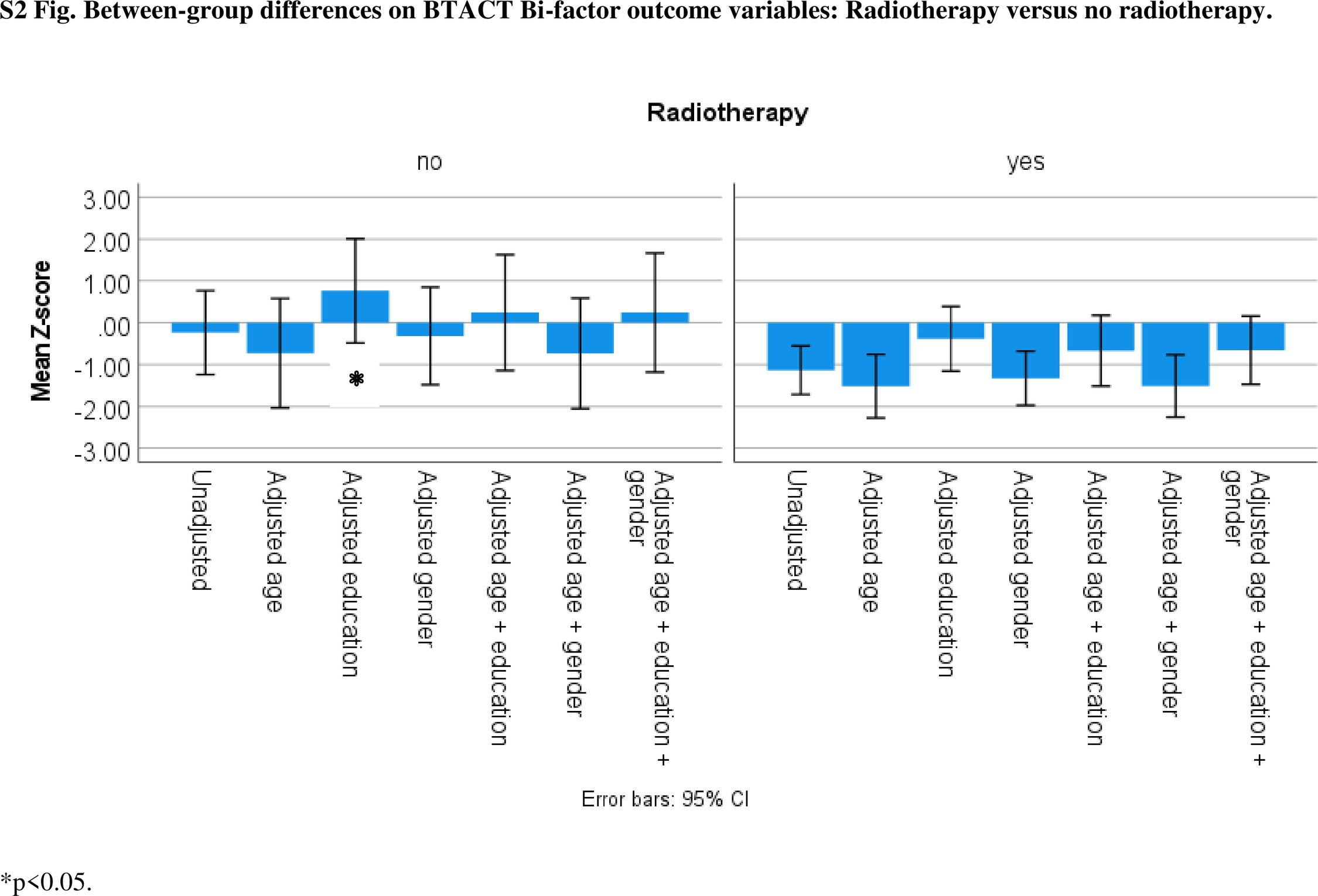

Supplement: S2 Fig — (TIF) [file pone.0296387.s002.tif]
