# Supplementary material for: Sleep and cognition in South African patients with non-functioning pituitary adenomas
Source: PLoS One. 2024 Jan 18;19(1):e0296387. doi: 10.1371/journal.pone.0296387 (PMC10796019; doi:10.1371/journal.pone.0296387)
Supplement: Attachment — Submitted filename: Response to Reviewers.docx [file pone.0296387.s003.docx]

19/09/2023

The Editor

PLoS one

**Regarding**: Sleep and cognition in South African patients with non-functioning pituitary adenomas

On behalf of my co-authors we thank you most sincerely for the review and suggestions, which we feel will improve the manuscript considerably. We have adopted all the suggestions and we believe that the manuscript is now ready for publication.

Editor’s comments:

In line with the editors comments:

A major point has to do with the process followed during the selection of the participants in the patient group and the inclusion/exclusion criteria, because several individuals appear to have other major health issues that may in fact affect cognitive function.

*We agree with this and as such we have removed the patients with epilepsy and HIV and included a statement in the methods section.*

**In view of the possibility that HIV and epilepsy may have an impact on cognitive function, we have excluded these two subjects.**

Moreover, given the very small sample size, the decision to split the patient group into two subgroups may create problematic issues with the statistical analysis therefore apart analysis for each separate statistical analysis also be provided to establish whether the sample size was sufficient for at least medium effect size be observed.

*We agree that, with regard to the secondary analyses especially, the study is underpowered. However, as those secondary analyses are merely exploratory, we elected to retain the description of their results while acknowledging that they must be interpreted cautiously. We have also included the following sentence in the Limitations subsection:*

**While the secondary analyses are underpowered, we feel the exploratory results may be useful to provide the impetus for future research.**

Please include your full ethics statement in the ‘Methods’ section of your manuscript file. In your statement, please include the full name of the IRB or ethics committee who approved or waived your study, as well as whether or not you obtained informed written or verbal consent. If consent was waived for your study, please include this information in your statement as well.

*We have now included the ethics approval in the methods section*.

**Approval for the study procedures was granted by the Human Research Ethics Committees of the Department of Psychology (PSY 2020-029) and Faculty of Health Sciences Research and Ethics Committee (HREC REF: 462/2019) of the University of Cape Town. The study was performed in accordance with the ethical standards as laid down in the 1964 declaration of Helsinki and its later amendments or comparable ethical standards.**

In your Data Availability statement, you have not specified where the minimal data set underlying the results described in your manuscript can be found. PLOS defines a study's minimal data set as the underlying data used to reach the conclusions drawn in the manuscript and any additional data required to replicate the reported study findings in their entirety. All PLOS journals require that the minimal data set be made fully available. For more information about our data policy, please see <http://journals.plos.org/plosone/s/data-availability>.

*We have provided a ZivaHub link, where the data can be accessed.The data link is as follows:*

[*https://figshare.com/s/f31b8954618a5e801532*](https://figshare.com/s/f31b8954618a5e801532)

*Reviewer 1*

*We thank the reviewer for his/her helpful comments in improving the overall quality of the manuscript.*

*Major points*

*1. In the introduction section there is no need to provide statistical results from these studies it should be modified.*

*We agree there is no need to provide those results in this section. Hence, we have removed the statistics and have changed the sentence to read as follows:*

**They found a significant association between sleep quality and cognitive performance (previously predicted worse cognitive performance) and concluded that the link between the two patients with pituitary disease warrants further investigation.**

2. In my opinion, there is a lack of general justification for the hypotheses . I appreciate that authors describe the limitations of the Wennberg et al. (2019) study. However, they should provide more general reasons for the aims of their study. Perhaps based on the gaps from other studies and/or neuropsychological [MH2] mechanisms of importance pituitary role for cognition

*We thank the Reviewer for this comment. We have addressed it by adding the following sentences a on the general aims of the study to the subsection titled “The Current Study”:*

**As explained in the review above, the pituitary gland is a central component of the neuroendocrine system that, via thyroid and glucocorticoid hormones, regulates the sleep-wake cycle and plays a critical role in memory processing. Hence, we wished to determine whether patients with non-functioning pituitary adenomas [NFPA] would present with compromised sleep quality and impaired cognitive performance, even when adequately replaced with hormone therapy. We also wished to determine, given known relationships (described above) between sleep and cognition, whether disrupted sleep in those patients might partially account for their compromised cognitive functioning.**

*3.* In my opinion, two patients may have cognitive dysfunctions as an impact of other diseases (HIV and epilepsy). I suggest to consider remove them and conduct a new statistical analysis.

*We have adopted the Reviewer’s suggestion elected to remove the two patients in question. We have rerun the analyses to determine whether removing these patients has an impact on the comparisons. Please see the revised Results section for the outcome of those analyses.*

Suggested references:

Woods, S. P., Moore, D. J., Weber, E., & Grant, I. (2009). Cognitive neuropsychology of HIV-associated neurocognitive disorders. Neuropsychology Review, 19, 152-168.

McCagh, J., Fisk, J. E., & Baker, G. A. (2009). Epilepsy, psychosocial and cognitive functioning. Epilepsy Research, 86, 1-14.

*We thank the reviewer for these references. They are now included in our reference list*

4 Did authors check the normal distribution of the analysis variables (S-W test, sAs in our methods section, we make use of the S-W test, skewness and kurtosis.

We agree with the Reviewer that these steps should be taken before undertaking any inferential statistical analyses. We indicate in the Data Management and Statistical Analysis section that we did indeed take these steps (see subsection titled Inferential Analyses). At that point in the manuscript we also added the following sentence to confirm that we checked the relevant statistical assumptions: our Results section:

**Our findings were such that we did not need to use nonparametric statistical comparisons.**

5. I strongly suggest using correction for multiple comparisons (e.g., Holm-Bonferroni or another) and adding a p-value after correction to the results.

*We agree with this comment and in view of the multiple comparisons we have rerun the statistical analyses utilising the Holm-Bonferroni correction for multiple comparisons. We have altered all the comparisons, taking into consideration this correction factor. We have also as suggested adding the p value after correction for these results. We have also included the following statement in the Data Management and Statistical Analyses section:*

**Given that our analyses featured multiple comparisons, we applied the Holm-Bonferroni correction factor and adjusted the P-value accordingly. Specifically, to correct for running 16 different analyses we set the significance level at p = 0.05/16 = 0.003.**

6. I suggest considering using a bootstrapping method to improve the statistical analysis of comparisons and relationships.

*We thank the Reviewer for this suggestion t. We performed the bootstrapping method for all analyses, using 1000 replicates and edited the Results section to reflect the changes.*

*We also now include the following statement in the Data Management and Statistical Analyses section:*

**We performed the bootstrapping method using 1000 replicates.**

7. I strongly suggest presenting some of the results in the Figures as the box [MH1] or violin plots (e.g., for comparison analysis).

Again, w*e thank the Reviewer for this suggestion. We now use figures to explicate results of the secondary analyses. These figures are now included as supplementary material.*

8. In the discussion section authors should add propositions for clinical practice.

*We agree that this is an important addition. We now include the following sentences in the Summary and Conclusion subsection of the Discussion.*

**This result not only contributes to scientific understanding of cognitive impairment in pituitary disease, but also has some significant clinical implications. For instance, it suggests that health professionals treating these patients must have a keen awareness of the possibility that cognitive difficulties may impact on treatment adherence and outcomes. Further, it may be important for those professionals to enquire from significant others about possible cognitive impairment, given that patients themselves may not have insight into their difficulties.**

**Another clinical implication is that patients with NFPAs may benefit from cognitive remediation alongside their usual medical care. Such remediation, which often consists of learning from computer-based games and using tangible everyday objects to stimulate cognitive processing, might assist in improving their memory function, a key component of medication adherence and other instrumental activities of daily living.**

9. In my opinion, there are much more limitations of this study. First, the use of neuropsychological tests during a phone call raises controversy, e.g., lack of control over potential distractors (noise, room conditions, and presence of other people during the test) or hearing impairments in the subjects. It is the main limitation of this study and authors should discuss it. Second, there is no information about the IQ of the participants and any methods to measure it. Third, only verbal forms of neuropsychological tests were used in the study. So, we can't interpret the motor aspects of cognitive functioning e.g., cognitive flexibility or motor control. It should be discussed. Finally, tasks used in this study are characterized by low "ecological validity". Please discuss the excellent work by Chaytor & Schmitter-Edgecombe (2003).[

Suggested reference:

Chaytor, N., & Schmitter-Edgecombe, M. (2003). The ecological validity of neuropsychological tests: A review of the literature on everyday cognitive skills. Neuropsychology Review, 13, 181-197.

*We thank the Reviewer for this insight. To address this comment, we added the following statement in the Limitations subsection of the Discussion:*

**Fourth, there are limitations related to the mode and form of cognitive assessment we used. Although administration of the BTACT (and similar telephone-based or online assessments) is valuable in ensuring participants can be reached easily and conveniently, such administration (a) offers relatively little control over potential distractors such as external noise and the presence of others in the room during assessment, (b) might be more negatively affected by participants’ hearing impairment than might in-person assessment administration, (c) is limited to the verbal modality only, meaning cognitive domains such as visuospatial memory and psychomotor coordination remain unassessed. Additionally, it might be argued that interpretation of the participants’ cognitive performance is affected by the lack of information about their overall IQ and by the fact that the cognitive tasks used in the study were of low ecological validity (see, e.g., Chaytor & Schmitter-Edgecombe (2003).**

Minor points:

1. The text should be checked for mistakes (e.g., in the text and Tables "p" should be written in lowercase).

*We have checked thoroughly for these and other mistakes in the text.*

2. There is no need to use stars when authors provide a p-value.

*We understand the reviewer's preference and hence we have removed the asterisks as superscripts to p-values.*

3. Is ESE the value of Cohen's d? It should be explained.

*In Table 3 ESE (Effect Size Estimate) is the result of the Cohen’s d calculation. We now state as much in the Note to Table 3.*

Reviewer 2

1. Please add detailed inclusion and exclusion criteria in the Methods section. The author mentioned excluding people with previous cognitive impairment, how was cognitive impairment identified here?

*We thank the reviewer for this comment. We have included the following sentence in the Method section:*

*Patients with NFPA were excluded from participation if they had a medical comorbidity (e.g., HIV, epilepsy; see, e.g., McCagh, Fisk, & Baker, 2009; Woods et al., 2009) that is known to have independent effects of cognitive functioning.***.**

2. On page 7, line 173, the author mentioned that people with diseases that may affect cognitive function and sleep quality were excluded. Please describe in detail which diseases were included.

**After excluding comorbidities known to adversely affect cognitive function and sleep quality, we included patients with the following comorbidities: diabetes and hypertension, which were deemed unlikely to have had material impact.**

3. On page 12, line 282, please add the full name when the abbreviation appears for the first time, such as M, SD. At the same time, the full names of M and SD should be added to the notes in Table 1 and Table 2.

*For clarity, we have defined M as mean and SD as standard deviation in the notes to Table 1 and Table 2*

4. In regression models with the GSAQ score and multiple BTACT scores as outcomes, were other covariates considered? Age, education, and gender appeared to be considered in the model with the Bi-factor scoring method for the BTACT outcome.

*Other factors were not considered in the mediation analysis because we were unable to complete the mediation analysis – sleep did not differ between patients and controls. In addition, we did not have the requisite power to control for these covariates. However, as mentioned by the Reviewer these covariate factors were controlled for by the BTACT bi-factor scoring method.*

Reviewer 3

- Overall, a series of statistical comparisons and correlations have been made, but it is unclear whether the authors corrected for multiple comparisons. If not, the type I error (false positive) rate can be inflated, possibly leading to erroneous inferences. I would strongly recommend correcting for multiple comparisons using an appropriate method and reporting transparently when the results are corrected/uncorrected (e.g., including this in the tables).

Please see our response to Reviewer 1’s Comment 5. **.**

- Furthermore, the rationale for the median split analyses separating the participants into ‘good’ and ‘bad’ sleepers is unclear, especially given the low sample size (N=10 and N=9). This could also contribute to the multiple comparisons problem. Please provide a clear rationale for these analyses, and report whether the results are corrected for multiple comparisons.

*We thank the reviewer for this question. We divided patients into good and bad sleepers based on the cut-offs recommended by developers of the Global Sleep Assessment Questionnaires (GSAQ). We also, as noted above, corrected for multiple comparisons using the Holm-Bonferroni correction factor. We now include the following statement in the Methods section:*

**We delineated patients into good and bad sleepers, based on the control groups median GSAQ score since there are no recommended cut-offs for the GSQA.**

- Given that subjective and objective sleep is often not correlated, this study may not have been sufficiently sensitive to capture sleep disruption. The authors acknowledge this in the discussion. However, the abstract is rather misleading in saying that the patients’ sleep quality is intact. In the absence of evidence for the null (e.g., equivalence testing, Bayesian, etc.), I would recommend modifying the abstract to better reflect their conclusions drawn in the main text.

*We acknowledged that the sleep quality of this sample of participants may be difficult to ascertain given the relative insensitivity of the instrument we used to measure that construct. We considered that the spectrum of sleep disturbance may be compared between patients and controls acknowledging the lack of objectivity. We agree with the Reviewer that the perception of what is a good night’s sleep may be distorted with having a pituitary adenoma and now acknowledge this fact in the Discussion (see response to comment below). We have also altered the relevant sentences in the Abstract so that they now read thus:*

*Patients’ global cognitive performance was significantly worse than that of controls (p= 0.01). However, spectrums of sleep disturbance did not differ significantly between patients and controls.*

Furthermore, considering that the patients have been living with pituitary adenoma for several years, their perception of what is ‘a good night of sleep’ will likely be different to that of healthy controls. Consider adding this point to the discussion.

As noted above, w*e agree with this comment and hence have added the following sentence to the Discussion:*

*Third, It is possible that patients with pituitary adenoma may normalize their sleep disturbances over time and hence may fail to recognize that they do indeed experience impaired sleep quality. In short, assessment of sleep quality using a self-report measure has significant shortcomings and it might have been preferable to assess this construct using objective measures.*

In the results section under ‘secondary analyses’, the authors infer that less sleep disturbance is likely associated with better cognitive performance than in the patients, however, the results are not significant. Did the authors compare the correlations? If not, please change the language accordingly to reflect a null result

*We find this comment particularly helpful and have compared correlations between sleep disturbance and cognitive impairment in patients and controls. We now stateexplicitly in the Results section that these differences did not reach statisical significance.:*

- I was unable to find the dataset using the link provided and when searching for it on ZivaHub. Please provide a correct link.

*We have provided a ZivaHub link where the data can be accessed.The link is:* [*https://figshare.com/s/f31b8954618a5e801532*](https://figshare.com/s/f31b8954618a5e801532)

Minor points

The page numbers are not accurate, please correct.

*We have corrected the page numbers.*

Page 3, lines 71-72: “… characterized by deep sleep and slow wave sleep (SWS on electroencephalogram readings”. I presume the authors meant that stage 3 is characterized by deep sleep or slow wave sleep. Also, the stages are characterized by polysomnography, usually meaning EEG as well as EOG and EMG.

*We thank the Reviewer for picking up on this lack of clarity in the original manuscript. We have made the appropriate corrections in the revised manuscript, so that the sentence in question now reads as follows:*

**The latter is further subdivided into three stages: stage 1 (light sleep), stage 2, and stage 3, which is characterized by deep sleep and slow wave sleep (SWS) on electroencephalogram, electroencephalogram and electromyography readings.**

Page 4, middle paragraph: With the concluding sentence suggesting that sleep is important for consolidation on subsequent days, it is unclear how this relates to the present study, considering that there was only one measure of episodic memory on the same day. Consider providing a clearer link to the present question.

Memory consolidation processes occurring during sleep may be similar to those occurring during waking (see, e.g., Siegel, 2021; Wamsley, 2019), although as reflected by our review there are many more studies of sleep-dependent consolidation than waking consolidation. Nonetheless, we take the Reviewer’s point that our exclusive citing of literature on sleep-dependent consolidation is not, at first glance, directly related to the design of the current study. We have added some text to the relevant part of the Introduction in an attempt to clarify the links between our study and the literature on memory consolidation.

Page 6, first paragraph: When introducing the previous similar study, it would be helpful to include which measures of sleep and cognition were administered for ease of comparison. This could also be reiterated when discussing the limitations of the sleep measure in the discussion.

We have added the relevant information to the paragraph in question.

Our sincere thanks indeed.

Dr Michelle Henry
