# Supplementary material for: Sleep and cognition in South African patients with non-functioning pituitary adenomas
Source: PLoS One. 2024 Jan 18;19(1):e0296387. doi: 10.1371/journal.pone.0296387 (PMC10796019; doi:10.1371/journal.pone.0296387)
Supplement: Attachment — Submitted filename: Reviewers new comments_final.docx [file pone.0296387.s004.docx]

06/12/2023

The Editor

PLoS one

**Regarding**: Sleep and cognition in South African patients with non-functioning pituitary adenomas

On behalf of my co-authors we thank you most sincerely for the review and suggestions, which we feel will improve the manuscript considerably. We have adopted all the suggestions and we believe that the manuscript is now ready for publication.

Reviewer Comments

Major points

**Updated concern:**
While the Authors have addressed my concern and corrected for multiple comparisons, this has had no bearing on their interpretation of the results in the abstract, discussion, and conclusion. After applying their correction methods, they did not find any significant differences, however, this is not reflected in the overall tone and take-home messages of the paper. I would recommend highlighting this in the relevant sections (abstract and conclusion).

**Updated Response:** We have edited the abstract, discussion and conclusion to better reflect the results.

We have added the following sentence to the abstract:

We found no significant differences in cognition between patients and controls after adjusting for multiple comparisons.

We have changed the sentence in the abstract to read:

Our data suggest that NFPA patients’ cognition and sleep quality is relatively intact, and that sleep disruption does mediate the cognitive

We have also made multiple edits to the discussion and conclusion to better reflect our non-significant findings (see tracked changes manuscript).

**Updated concern:** In the table notes, there is no mention of whether the p-values are corrected for multiple comparisons or not. Please include this on all table notes.

**Response:** P-values were not corrected for multiple comparisons, but were only considered significant if p was less than 0.003 to allow for multiple comparisons. We have mentioned this in the statistical analyses section of the paper and added this note to Tables 3-5.

**Updated concern:**
My concern was not with the choice of cut-off threshold, but rather with why they separated the group in the first place, i.e., what was the rationale that made them split the group into good and bad sleepers. Considering the small sample size, I think there needs to be a solid reason for this analysis.

**Response:** We have removed this analysis from the paper.

Minor points

 **Updated concern:**
While I appreciate that the Authors want to draw a parallel between the literature on memory consolidation during wake and sleep, these two paragraphs (on memory consolidation during sleep and wake) seem to make the rationale for looking at sleep disruption and memory even more confusing. Apologies if I was unclear when I recommended that the Authors make the rationale clearer. The key discrepancy between the literature they are referring to and their study is that this study is not measuring memory across a period of sleep, but rather whether poor sleep is linked to poor memory formation. What might help the rationale is to focus less on sleep-associated memory consolidation and instead focus on the literature which has found that sleep disruption prior to learning is detrimental for memory formation (for example, see this meta-analysis: <https://doi.org/10.1037/bul0000348>). The Authors have included a sentence on this, but the take-home message of that paragraph seems to be that sleep disruption after learning is detrimental for consolidation.

**Response:** We have edited the paragraph in the introduction to include more about sleep disruption prior to learning:

Research has shown that healthy sleep supports memory functioning in two ways: a good night’s sleep helps prepare the brain for new learning the following day, and sleep-dependent memory consolidation helps strengthen new memories learned during the day (Newbury et al., 2021). Sleep plays an important role in restoring neuronal functioning for new learning on the subsequent day (Tononi & Cirelli, 2003). Several studies have confirmed this and shown that when sleep is deprived, memory encoding is disrupted (e.g., Alberca-Reina et al., 2015). Studies also suggest that an uninterrupted night of sleep prior to learning allows for more efficient retrieval of that information on subsequent occasions (Cordeira et al. 2018; Walker 2008).

Our sincere thanks indeed.

Dr Michelle Henry
